# Data set for extraction and transesterification of bio-oil from *Stoechospermum marginatum*, a brown marine algae

**DOI:** 10.1016/j.dib.2017.08.031

**Published:** 2017-08-30

**Authors:** Hariram Venkatesan, John J. Godwin, Seralathan Sivamani

**Affiliations:** a Department of Mechanical Engineering, Hindustan Institute of Technology & Science, Hindustan University Chennai, India; b Department of Automobile Engineering, Hindustan Institute of Technology & Science, Hindustan University Chennai, India

**Keywords:** Biodiesel, Marine algae, Transesterification, Molar ratio

## Abstract

The article presents the experimental data on the extraction and transesterification of bio-oil derived from *Stoechospermum marginatum,* a brown macro marine algae. The samples were collected from Mandapam region, Gulf of Mannar, Tamil Nadu, India. The bio-oil was extracted using Soxhlet technique with a lipid extraction efficiency of 24.4%. Single stage transesterification was adopted due to lower free fatty acid content. The yield of biodiesel was optimized by varying the process parameters. The obtained data showed the optimum process parameters as reaction time 90 min, reaction temperature 65 °C, catalyst concentration 0.50 g and 8:1 M ratio. Furthermore, the data pertaining to the physio-chemical properties of the derived algal biodiesel were also presented.

**Specifications Table**

**Table:** 

**Subject area**	Alternate fuels
**More specific subject area**	Biodiesel, 3rd generation bio-fuel
**Type of data**	Figures and Tables
**How data was acquired**	Experimental analysis in laboratory
**Data format**	Raw and Tabulated
**Experimental factor**	Yield of biodiesel and its Physiochemical properties
**Experimental features**	Transformation of marine seaweed into bio-fuel and analysis of its physio-chemical properties
**Data sources**	Fuels and lubricants laboratory, Hindustan University
**Data accessibility**	Data is with this article

**Value of the data**

• This data provides a methodology for converting marine seaweed into biodiesel, value added product.
• The data's tabulated describes the best possible way to obtain maximum yield of biodiesel through optimizing reaction time, reaction temperature, catalyst concentration and molar ratio.
• The data presented here also details the physio-chemical properties of the yielded biodiesel and its suitability as engine fuel.

## 1 Data

**Fig. 1:**
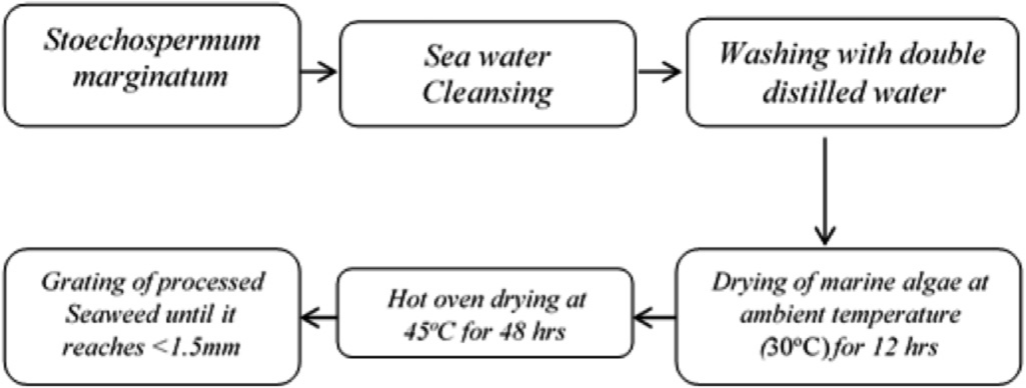
Pre-treatment procedure for marine algae.

**Fig. 2:**
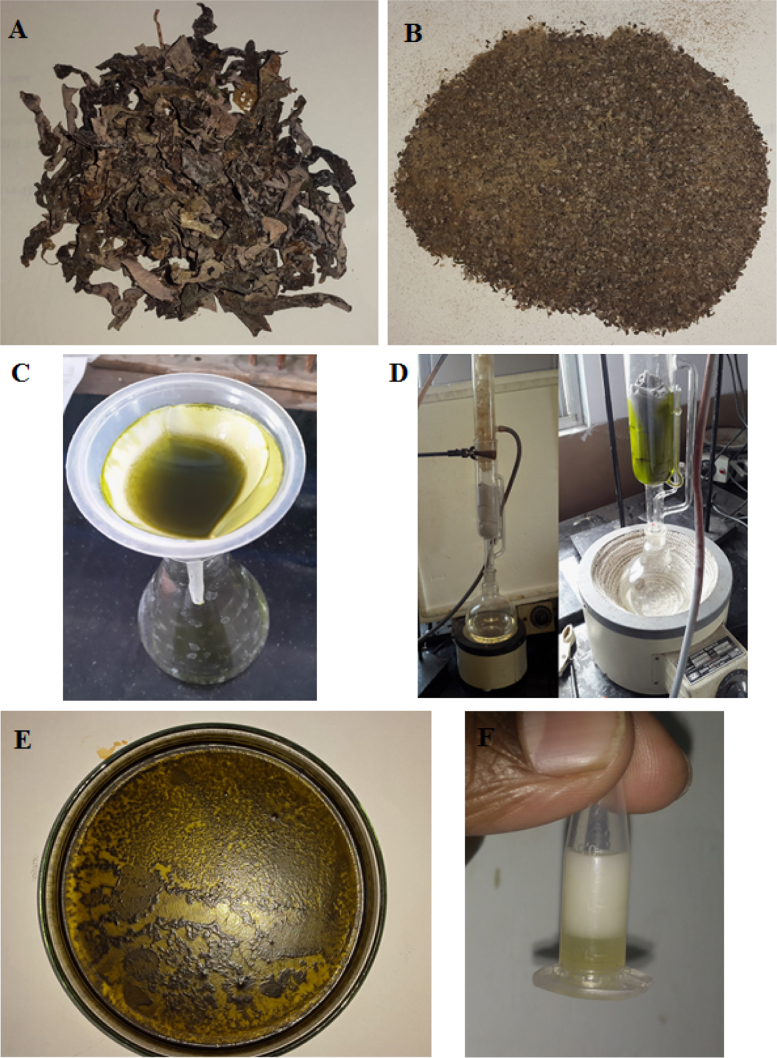
Synthesis and transesterification of bio-oil through maceration and Soxhlet procedure from *Stoechospermum marginatum,* a brown seaweed.

Fig. 1 shows the pre-treatment procedure for marine algae for extracting bio-oil. Fig. 2 illustrates the extraction and transesterification of bio-oil from *Stoechospermum marginatum.* Table 1 describe the yield of biodiesel by optimizing the various process parameters like reaction time, reaction temperature, catalyst concentration and molar ratio. Table 2 illustrates the physio-chemical properties like density, kinematic viscosity, calorific value, cetane number, flash and fire point of transesterified bio-oil from *S. marginatum*.

**Table 1:** Process parameters for optimized biodiesel yield.

**Reaction time (in min)**	**Reaction temperature (in °C)**	**Catalyst concentration (in g)**	**Molar ratio**	**Yield of biodiesel (in %)**
70	55	0.42	6:1	40.52
70	60	0.45	7:1	42.12
70	65	0.50	8:1	48.43
70	70	0.54	9:1	32.14
80	55	0.42	6:1	67.54
80	60	0.45	7:1	69.04
80	65	0.50	8:1	70.12
80	70	0.54	9:1	37.25
90	55	0.42	6:1	56.81
90	60	0.45	7:1	54.12
90	65	0.50	8:1	72.16
90	70	0.54	9:1	40.37
100	55	0.42	6:1	53.43
100	60	0.45	7:1	60.05
100	65	0.50	8:1	69.89
100	70	0.54	9:1	41.75

**Table 2:** Physio-chemical properties of transesterified bio-oil derived from *Stoechospermum marginatum*.

**Properties**	**ASTM D975**	**ASTM 6751**	**Mineral diesel**	**Algal biodiesel**
Density at 15 °C (kg/m^3^)	–	860–900	850	890
Kinematic viscosity at 40 °C (mm^2^/s)	1.9–4.1	3.5–5.0	2.6	4.84
Calorific value (kJ/kg)	–	–	44,800	42,052
Cetane number	40	47	46	63
Flash point (°C)	52	101	64	128
Fire point (°C)	–	–	70	136

## 2 Experimental design, materials and methods

### 2.1 Materials

*Stoechospermum marginatum*, a brown seaweed was collected from the inter tidal region of Mandapam, Ramanathapuram district, Tamil Nadu, India. 99.9% pure industrial grade *n*-hexane was purchased from Mivion Chemicals, Mumbai, India. Industrial grade NaOH and Methanol was supplied by Accord Chemical Corporation, Mumbai, India. The other materials used for oil extraction and transesterification were Erlenmeyer flask, flat and round bottomed flask and side arm flask and soxhlet apparatus.

### 2.2 Methods

The collected seaweed was cleansed thoroughly with sea water to remove the adhered sand followed by washing with double distilled water. The brown seaweed was brought to the laboratory and dried for 12 h at ambient temperature under sunlight. For further removal of moisture, the seaweed was placed in a hot oven at 45 °C for 48 h. The processed seaweed was grated to reach the size less than 1.5 mm in a pestle grinder as indicated in Fig. 1. Maceration and Soxhlet techniques were adopted to extract the bio-oil [4].

### 2.3 Bio-oil extraction methods

In maceration technique, 50 g of processed seaweed was soaked with 500 ml *n-*hexane in a round bottomed conical flask and maintained at 45 °C as shown in Fig. 2. The chemical reaction ruptured the cell wall membrane of the algae expelling lipids. The reaction period of 72 h in a closed chamber yielded 4.5 ml of lipid at an efficiency of 9.2% [1].

In Soxhlet extraction technique, the thimble was filled with 15 g of processed seaweed. The bottom layer of the extractor is filled with 500 ml *n*-hexane solution. On heating upto 70 °C, the *n*-hexane vaporises and reaches the top layer of the thimble through the side arm. The condensation arrangement liquefies the *n*-hexane vapour and reacts with the algal biomass rupturing the cell wall. The expelled lipid mixes with *n*-hexane forming a colloidal solution and is brought down to the round bottomed flask. This cycle was repeated 10 to 12 times which collected the lipid from the algal biomass into the round bottomed flask as shown in the Fig. 2D. The maximum yield of the bio-oil was found to be 3.6 ml/15 g algal biomass at an extraction efficiency of 24.4% [3].

On comparing both extraction methods, Soxhlet extraction technique was found to be more efficient. 95% of *n*-hexane was recovered during the above process.

### 2.4 Transesterification of bio-oil

Single stage transesterification process was adopted due to free fatty acid content being less than 1.7%. The process parameters like reaction time, reaction temperature, catalyst concentration and molar ratio were optimized to yield maximum quantity of algal biodiesel. Based on the literature survey, the reaction time was varied between 70 and 100 min, reaction temperature between 55 °C and 70 °C, NaOH concentration between 0.4 and 0.54 g and molar ratio between 6:1 and 9:1. The maximum yield of biodiesel was noticed to be 72.16% at 90 min reaction time, 65 °C reaction temperature, 0.50 g catalyst concentration and 8:1 M ratio as shown in Table 1.

### 2.5 Physio-chemical properties

Kinematic viscosity is an important property to understand its resistance to flow under the laminar flow principle. 50 ml of algal biodiesel was filled in the oil cup and its temperature is elevated upto 40 °C by the surrounding water bath. Uniform thermal equilibrium was attained by stirring action of the algal biodiesel. At 40 °C the flow jet was opened allowing algal biodiesel to flow into the kohlrausch flask to estimate the kinematic viscosity of algal biodiesel and it was found to be 4.84 mm^2^/s [2].

Flash and fire point is an important physio-chemical property to understand the flammability limits of liquid fuels. The test was carried out as per ASTM D93 standards using Pensky marten closed cup apparatus. 50 ml of test fuel was filled in the oil cup. The temperature was elevated using the gas burner and thermal equilibrium was maintained with the help of mechanical stirrer at 60 rpm. The rise in temperature was recorded at an interval of 1 °C above 85 °C. The flash and fire point of algal biodiesel was noticed to be 128 °C and 136 °C respectively.

The calorific value indicates the total amount of heat energy which can be produced by a unit quantity of liquid fuel on complete combustion. The gross calorific value of algal biodiesel was identified through ASTM D5865 using bomb calorimeter. 20 ml of algal biodiesel was placed in the crucible and the lid was closed tightly. Excess oxygen was supplied at the pressure of 25 to 30 atm. On passing electric current, the combustion of fuel inside the bomb calorimeter along with its temperature difference was used to calculate the calorific value. The gross calorific value of algal biodiesel was found to be 42,052 kJ/kg.

Cetane number is an important property which indicates the ignition quality of the liquid fuel. It is was calculated by means of tabulating the cetane indices using ASTM D976. The calculated cetane number of algal biodiesel was noticed to be 63.

ASTM D4052 standards was used to estimate the density of algal biodiesel. Densometer was used to calculate the density of algal biodiesel. The density of the algal biodiesel was found to be 890 kg/m^3^ [2], [5].

## References

[1] Grima M.E., Robles Medina A., Gimenez Gimenez A., Sanchez Perez J.A., Garcra Camacho F., Garcra Sanchez J.L. (1994). Comparison between extraction of lipids and fatty acids from microalgal biomass. J. Am. Oil Chem. Soc..

[2] Hariram V., Vasanthaseelan S. (2016). Characterization of FAME's in canola biodiesel using spectroscopic studies. Int. J. Chem. Sci..

[3] Chisti Y. (2007). Biodiesel from microalgae. Biotechnol. Adv..

[4] Murugaiyan K., Narasimman S., Anatharaman P. (2012). Proximate composition of marine macro algae from Seeniappa Dharka, Gulf of Mannar region, Tamil Nadu. Int. J. Res. Mar. Sci..

[5] Hariram V., Mohan Kumar G. (2013). Combustion analysis of Algal oil methyl ester in a direct injection compression ignition engine. J. Eng. Sci. Technol..

